# Seaweed-Based Compounds and Products for Sustainable Protection against Plant Pathogens

**DOI:** 10.3390/md19020059

**Published:** 2021-01-25

**Authors:** Pushp Sheel Shukla, Tudor Borza, Alan T. Critchley, Balakrishnan Prithiviraj

**Affiliations:** 1 Marine Bio-Products Research Laboratory, Department of Plant, Food and Environmental Sciences, Faculty of Agriculture, Dalhousie University, Truro, NS B2N5E3, Canada; pushpsheel.shukla@dal.ca (P.S.S.); Tudor.Borza@dal.ca (T.B.); 2 Verschuren Centre for Sustainability in Energy and Environment, Cape Breton University, Sydney, NS B1M1A2, Canada; alan.critchley2016@gmail.com

**Keywords:** biostimulants, seaweed, bioactive compounds, plant pathogens

## Abstract

Sustainable agricultural practices increasingly demand novel, environmentally friendly compounds which induce plant immunity against pathogens. Stimulating plant immunity using seaweed extracts is a highly viable strategy, as these formulations contain many bio-elicitors (phyco-elicitors) which can significantly boost natural plant immunity. Certain bioactive elicitors present in a multitude of extracts of seaweeds (both commercially available and bench-scale laboratory formulations) activate pathogen-associated molecular patterns (PAMPs) due to their structural similarity (i.e., analogous structure) with pathogen-derived molecules. This is achieved via the priming and/or elicitation of the defense responses of the induced systemic resistance (ISR) and systemic acquired resistance (SAR) pathways. Knowledge accumulated over the past few decades is reviewed here, aiming to explain why certain seaweed-derived bioactives have such tremendous potential to elicit plant defense responses with considerable economic significance, particularly with increasing biotic stress impacts due to climate change and the concomitant move to sustainable agriculture and away from synthetic chemistry and environmental damage. Various extracts of seaweeds display remarkably different modes of action(s) which can manipulate the plant defense responses when applied. This review focuses on both the similarities and differences amongst the modes of actions of several different seaweed extracts, as well as their individual components. Novel biotechnological approaches for the development of new commercial products for crop protection, in a sustainable manner, are also suggested.

## 1. Introduction

Changing environmental and climatic conditions have the potential to increase the susceptibility of crops to numerous pathogens, i.e., biotic stress [1,2]. The proliferation of plant diseases greatly reduces crop yield and quality. Many pathogens produce toxins and, in so doing contaminate the produce after harvest, which can cause severe economic loss to crop production worldwide [3,4]. Whilst not all of the foregoing problems occur with all crops, all at the same time, nor is their occurrence entirely predictable, it is current agricultural practice to take an “insurance approach”. This relies on the use (sometimes over-usage) of numerous synthetic chemical pesticides to control plant pathogens; however, the extensive input of pesticides is harmful to the farmers, consumers as well as ecosystems [5].

Through the advent of techniques in molecular biology, transgenic crops which carry various disease-resistance genes have been obtained from distant, usually wild, relatives. However, the successful implementation of the limited number of crops within which disease resistance has been achieved, through genetic modification/insertion (i.e., GMO crops) will require regulatory clearance from government authorities. In general, global public opinion is not favorable to this approach [6]. Alternatively, sustainable strategies of induced biotic resistance in important cultivated plants can also be achieved by the application of natural elicitors which can be derived from various seaweeds [7]. Over time, plants have developed an inherent immune system in order to fight pathogen aggression [6]. Plants respond to pathogen infestations either by Systemic Acquired Resistance (SAR) or Induced Systemic Resistance (ISR). SAR determines hypersensitive responses against pathogen infestations and is mediated by salicylic acid and pathogenesis-related (PR) proteins [8].

Plants activate their resistance mechanisms against pathogens by recognizing pathogen-associated molecular patterns (PAMPs) [9]. Various PAMPs can bind to the pattern recognition receptors on the plant cell membrane including flagellin (the major protein of the bacterial flagellum) and complex polysaccharides, such as chitin or different glucans. The binding of PAMPs to the appropriate pattern recognition receptor on the plant cell membrane leads to the activation of downstream signalling events, which, ultimately, will trigger a defense response [10]. Elicitors are those compounds recognized as PAMPS and these trigger the induction of the expression of genes involved in defense responses. During evolution, various seaweeds developed their own efficient defense mechanisms, hence there is a paucity of epidemics of infectious disease in natural seaweed populations [11,12]. Seaweeds are integral inhabitants of global coastal ecosystems and provide important ecosystem services which sustain the biodiversity of the in-shore marine environment [12]. From ancient times, seaweeds were used as natural soil conditioners [13,14]. In more recent decades, the uses of many seaweeds have been expanded immensely, and are evolving as a new trend, with increasing prospects their usage, i.e., food and bioactive compounds within functional foods and feed ingredients, phycocolloid production, plant biostimulants, biofuels and bioremediation [15,16,17]. Polyphenols, such as phlorotannins, and carbohydrates such as carrageenan, laminarins, fucoidans and ulvans, may act as elicitors which, when applied, can induce immunity against various plant pathogens [12,18,19,20]. The presence of these bioactive compounds has drawn the interest of various agrochemical companies for the production of commercial biostimulants using the biomass of a selected number of seaweeds [20,21,22]. 

The various modes of actions of seaweed-derived biostimulants largely depends on the raw materials and the applied methods of extraction [20]. In this review, we discuss similarities and differences among the various modes of actions of selected extracts derived from seaweeds.

## 2. Bioactive Compounds Present in Different Seaweeds and Their Mode of Action

Marine macroalgae are one of the most abundant sources of various bioactive compounds and have been widely used in agriculture to induce plant defense against various pathogens [23]. Table 1 lists the reports published on bioactive compounds from seaweeds and their role in eliciting responses from plant pathogens. The polymers highlighted are as reported in the literature. It may be that these are not always purified and may well represent polymer-rich fractions. There could well be cross-contamination with other co-extracted, seaweed-derived polymers. This is an avenue for future research that we suggest needs consolidated attention, whereby the bioactivities of purified fractions from complex seaweed extracts should be examined rigorously in order to determine modes of action and cause and effect responses.

### 2.1. Alginates

Alginates are linear polysaccharides composed of alternating units of β-d-mannuronate and α-l-guluronate residues linked by 1,4 glycosidic linkages and are important constituents of the cell wall of marine brown algae [63]. Alginates are widely researched for their commercial uses in food products, agriculture, materials, medicine, and biological science [63,64]. The egg-box interaction of alginates with calcium ions is reported to increase their biological activity [65,66]. Alginates, as derived from seaweeds, are made up of various ratios of mannuronic (M) and guluronic (G) acids. These ratios differ (M:G) with the part of the algal thallus from where they are isolated and also determine the rheological properties of the commercial alginate extracts. However, the individual effects of either M and G or the M:G ratios to bioefficacy in plant responses are not known. Clearly, further work is called for. Alginates have been shown to exhibit bio-stimulatory activities and mitigate stress tolerance in plants [67,68,69,70]. They also act as PAMPs and their application induces the innate immune system of plants. Bouissil et al. [25] showed that sodium alginate, as isolated from *Bifurcaria bifurcata* and *Fucus spiralis*, induced natural defense responses in the roots of date palm (*Phoenix dactylifera*) by regulating PAL and polyphenol metabolism. The activity of PAL was significantly higher in roots of the date palm treated with alginate-based elicitors [25]. Dey et al. [24] showed that an application of sodium alginate to tomatoes reduced the progression of early blight disease, caused by *Alternaria solani*. The foliar application of alginates induced SAR by stimulating the accumulation of H_2_O_2_ in response to pathogen infection and by reducing the activity of catalase (CAT) involved in the scavenging of H_2_O_2_. In addition to this, the activity of superoxide dismutase (SOD) was found to be increased upon the application of sodium alginate in tomatoes [24]. In the same study, the transcript expression of the non-expressor of pathogenesis related protein 1 (*NPR1)*, β-1,3-glucanase (*PR2)*, lipoxygenase D (*LOXD)*, and ACC oxidase 1 genes involved in the defense signalling pathways was found to be increased in the alginate-pretreated seedlings. Similarly, sodium alginate induced the biosynthesis of stilbenes and flavonoids, which are known to play major roles in defense responses, in the cell suspension of *Vitis vinifera,* and which also induced the expression of genes involved in their biosynthesis, including that of *PAL*, cinnamate 4-hydroxylase, and 4-coumarate: CoA ligase, stilbene synthase and chalcone synthase [71]. The alginate-treated *V. vinifera* cell suspension also showed a higher activity of pathogenesis-related proteins (PR), such as chitinase and β-1,3-glucanase [71]. Oligoguluronates obtained after the acid hydrolysis of sodium alginate isolated from *Laminaria hyperborea* elicited defense responses against pathogenic bacteria inhabiting the surface of the thallus of *L. hyperborea* and an epiphyte *Laminariocolax tomentosoides* [72]. These oligoguluronates induced oxidative stress in *L. digitata* and, in response, high levels of iodide were released to scavenge ROS [72]. Alginate oligosaccharides (AOS), prepared by the enzymatic treatment of the alginates isolated from brown algae, induced the biosynthesis of phytoalexins in response to the *Magnaporthe grisea* infection in rice [73]. A foliar spray of 1 mg/mL of AOS reduced the incidence of rice blast disease from 17.7 to 10.8% by regulating the activities of defense responsive enzymes such as peroxidase (POD), CAT and PAL. To decipher the molecular action of AOS in plant defense, Zhang et al. [27] used *Arabidopsis thaliana* to evaluate AOS-induced resistance to *Pseudomonas syringae* pv. *tomato* DC3000. A pre-treatment with 25 mg/L of AOS reduced the disease symptoms by 35.6%, as compared to control. The authors proposed that the phenotype was due to the early induction of signalling molecules, such as ROS and nitric oxide (NO) in AOS-treated leaves [27]. The elicitation properties of AOS in inducing resistance was lessened in the *sid2* mutant plants impaired in the SA biosynthetic pathway. In addition to this, the transcript of SA-dependent PR1 was found to be significantly higher in AOS-treated plants [27]. These results suggest that AOS-pre-treatment elicited resistance in *A. thaliana* by activating the SA-dependent defense-signalling pathway and stimulated natural, endogenous systems by regulating defense-responsive signalling pathways.

### 2.2. Carrageenans

Carrageenans are linear, partially hydrophilic sulphated polygalactans composed mainly of alternating units of d-galactose and 3,6-anhydro-galactose, linked by α-1,3 and β-1,4-glycosidic linkages [12,74]. Based on the degree and position of the sulphate groups, carrageenans are classified into six main types: iota (ι)-, kappa (κ)-, lambda (λ)-, mu (μ)-, nu (ν)- and theta (θ)-carrageenans [12,75]. Kappa (κ)-carrageenans are mainly isolated from the red alga *Kappaphycus alvarezii* through the hot extraction process and consist of d-galactose sulphated at the C4 position, linked to anhydro-galactose [76]. Structurally, beta (β)-carrageenans are identical to the kappa (κ)-carrageenans but lack sulphate on the C4 of the 1,3-linked units [12]. λ-carrageenans are more hydrophilic than κ-carrageenans and consist of d-galactose, having the sulphate group at the C2 position linked to a d-galactose sulphated at the C2 and C6 positions. λ-carrageenans are extracted from red algae such as *Gigartina* and *Chondrus* using an alcohol precipitation process [12]. ι-carrageenans are composed of d-galactose having the sulphate group at the C4 position, linked to an anhydro galactose sulphated at the C2 positions, and are commercially extracted from the red alga *Eucheuma denticulatum* [76]. Variation in sulphate content (i.e., 20% κ-carrageenan, 33% ι-carrageenan, 41% λ-carrageenan) leads to differential biological activities. Carrageenans are widely used in a large number of commercial applications in food and dairy industries, drug delivery, and the pharmaceutical industry, including antiviral, antitumor, immunomodulatory, antihyperlipidemic and anticoagulant properties [12,75,77,78,79]. Carrageenans and their pre-cursor of hydrolytic cleavage products, the oligo-carrageenans (OCs), also elicit natural plant defense responses against pests and pathogens by modulating the activity of different defense pathways, including salicylic acid (SA), jasmonic acid (JA) and ethylene (ET) signalling pathways [12]. The level of sulphation of the types of carrageenans is suggested to influence their specific activities and, therefore, their targeted applications for plant defenses [12]. It should be stated that further research is required in order to determine their absolute specificities. Clearly this work is of commercial importance and should be viewed as urgently required.

Diseases caused by viroids cause important commercial losses in agriculture [12,80]. Viroids replicate in the nucleus or chloroplast and spread by moving from cell to cell via plasmodesmata. Sangha et al. [40] showed that λ-carrageenan changed the biochemical status of tomato plants, inducing the defense mechanisms against Tomato Chlorotic Dwarf Viroid (TCDVd) and thereby controlling viroid replication. λ-carrageenan-treated plants showed changes in the regulation of several genes, including up-regulation of genes such as lipoxygenase *(LOX)*, allene oxide synthase and *PR1*, suggesting a JA-mediated response in treated plants against TCDVd [40]. 

Carrageenans are reported to protect plants and animals from viral infections by limiting their binding to receptors and internalization of viral particles into the host cells [76,81,82,83]. Sulphated polysaccharide 4 (SPS4) extracted from the red alga *Hypnea musciformis* contains 98% κ-carrageenan [47]. The infiltration of tobacco leaves with 200 μM SPS4 significantly reduced the number of lesions caused by tobacco mosaic virus (TMV) infection by inducing the accumulation of secondary metabolites, such as sesquiterpenoid and scopoletin. SPS4 activated plant defense mechanisms against viral infection by controlling the expression of the genes involved in SA- and JA/ET-dependent signalling pathways. Gene expression analysis showed that *PR1a, PR2, PR5, PR3* and *Def1.2* (defensin protein) were upregulated in leaves infiltrated with SPS4, in response to TMV infection [47]. Nagorskaya et al. [84,85] reported on the antiviral activity of κ/β-carrageenan isolated from the red alga *Tichocarpus crinitus* against TMV and potato virus X particles. The detached leaf assay showed that κ/β-carrageenan from *T. crinitus* induced lytic processes in *Datura stramonium* by controlling the intracellular accumulation and translocation of potato virus X particles [84]. A foliar spray of κ-, λ-, or ι-oligo-carrageenans, at the concentrations of 0.5, 1 or 5 mg/mL, induced defense responses in tobacco plants, resulting in enhanced protection against TMV, *Pectobacterium carotovorum* and *Botrytis cinerea* by inducing PAL biosynthesis—an important enzyme involved in the regulation of secondary metabolism and defense-responsive signalling pathways [48]. This study showed that OCs induced a long-term protection against TMV, which was dependent on dose, time, and number of treatments, mimicking a vaccine-like action, particularly when λ-carrageenan was used. 

Carrageenans and oligo-carrageenans can also significantly reduce the progression of fungal and bacterial diseases [12]. κ-OCs prepared by the enzymatic hydrolysis of κ-carrageenan were found to elicit the activity of laminarinase involved in plant defense in the cells of *Rubus fruticosus* [86]. Mani and Nagarathnam [44] showed that κ-carrageenan isolated from *K. alvarezii* had antifungal properties against *Colletotrichum gloeosporioides* which causes anthracnose disease in *Capsicum annuum.* An in vitro assay revealed that κ-carrageenan inhibited the mycelial growth of *C. gloeosporioides* by increasing its plasma membrane permeability. A foliar spray of *C. annuum* with κ-carrageenan elicited defense responses by regulating the enzymatic activity of POD and the expression of SA-/JA-dependent genes. Detailed proteomic analyses revealed the induction of proteins involved in nitric oxide synthesis, pathogenesis-related protein production and phytoalexin synthesis, whilst gene expression analyses indicated that cyclin-dependent protein kinase *(CDPK), PR1* and *NHO1* were also up-regulated [46]. Amongst other carrageenans, λ-carrageenan was found to be the most potent elicitor because of its high sulphur content, inducing systemic resistance against *P. parasitica* var. *nicotianae* in tobacco cells [44]. The induced resistance found in the tobacco cells was due to the higher expression of *sesquiterpene cyclase*, involved in the synthesis of the phytoalexin capsidiol, a functional chitinase coded by *PR3* genes. In addition to this, the cellular SA, and the transcripts of lipoxygenase *(LOX)* and ACC oxidase *(ACO)*, involved in JA- and ET-biosynthesis, were also found to be upregulated in λ-carrageenan-treated cells [44]. Pettongkhao et al. [43] showed that the leaves of the rubber tree (*Hevea brasiliensis*) sprayed with 0.5 mg/mL of λ-carrageenan, as isolated from the red alga *Acanthophora spicifera* stimulated immunity against *Phytophthora palmivora* by inducing the expression of SA-dependent defense responsive genes, which was further substantiated by the higher accumulation of SA and scopoletin. The activity of catalase, involved in ROS-scavenging, was suppressed in λ-carrageenan-treated rubber tree leaves, whilst POD activity was induced in treated leaves. The plausible explanation to these expression patterns is that the higher SA content in treated rubber leaves might have inhibited catalase activity [87], while the induction of *peroxidase* stimulated plant defense by regulating the process of lignification [88,89]. Le Mire et al. [45] showed that a foliar spray of λ-carrageenan reduced the progression of *Septoria tritici* blotch (STB) disease in wheat, caused by the fungal pathogen *Zymoseptoria tritici,* by inducing both SA- and JA-dependent signalling pathways. In another report, λ-carrageenan was reported to elicit a JA-dependent defense response in *A. thaliana* against *Sclerotinia sclerotiorum* by inducing the expression of the JA-induced, defense-related genes, such as *AOS*, *PDF1.2* and *PR3* [42]. *S. sclerotiorum* infected the plant by producing oxalic acid, which reduced local defense responses by the treated plants against the pathogen. Sangha et al. [42] reported that λ-carrageenan suppressed the *S. sclerotiorum*-mediated accumulation of oxalic acid by inducing in planta oxalate oxidase activity. The complementation assay using an *A. thaliana* mutant showed that the defense-eliciting activity of λ-carrageenan was observed in the salicylic acid-deficient mutant *ics1 but* did not rescue the susceptibility of *jar1* plants from *S. sclerotiorum* infection. However, ι-carrageenan-treated plants showed an increased susceptibility towards *S. sclerotiorum* infection [42].

Taken together, these results indicate that carrageenans and their oligomers, as derived from a variety of red seaweeds, are a very important source of bioactive compounds, eliciting the natural defense system and conferring resistance against a wide range of broad-spectrum pathogens in treated plants. 

### 2.3. Laminarins

**Figure 1:**
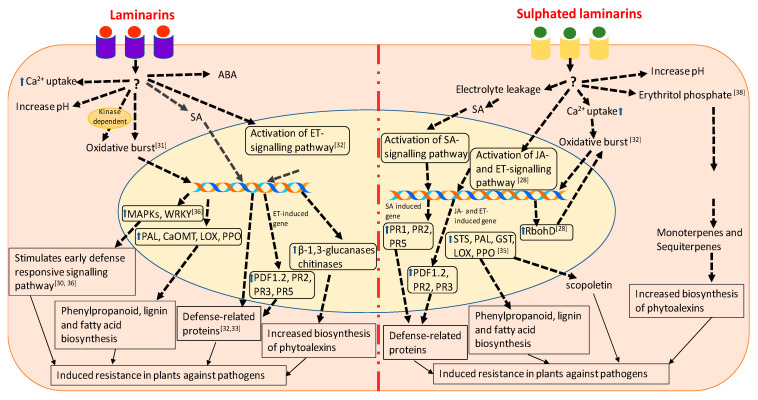
Schematic representation of the cellular functions and signalling pathways involved in defense mechanisms elicited in laminarin and sulphated laminarin. The drawing represents a synthesis of the work described by [28,31,32,33,34,35,38].

Laminarins (alt. spelling laminarans) are important constituents of the cell walls of brown seaweeds and provide flexibility to those algal thalli to withstand the pressure exerted by hydrodynamic forces [90,91,92,93]. Laminarins are generally low molecular weight (e.g., 5 kDa) storage β-glucans comprising (1,3)-β-d-glucans, having (1,3)-β-d-glucopyranose residues with some 6-*O*-branching in the main chain and some β-(1,6)-intrachain links [90]. Various published reports have found laminarins to possess biostimulant properties, and they are used extensively as immunostimulants, antitumour agents, anticoagulants, and as wound-healing agents in pharmaceutical, and cosmetic industries [92,93]. Laminarins also possess antioxidant and antimicrobial activities [90,94,95]. Currently, Iodus and Vacciplant (Arysta LifeScience, Cary, NC, USA) are products derived from laminarins that have been commercialized in various countries in order to control powdery mildew in strawberry and cereals, bacterial fire blight on apple trees and the grey mould in grapevine [39,96,97]. Foliar sprays of laminarins extracted from *Laminaria digitata* reduced the dependence of fungicides for the control of grey mould and powdery mildew in strawberry [29]. Similarly, Pugliese et al. [34] showed that the foliar application of laminarins reduced the natural incidence of powdery mildew in strawberry caused by *Erysiphe necator*. Laminarins extracted from *L. digitata* elicited the defense response in tobacco cells in a dose-dependent manner [31]. A treatment with 200 μg mL^−1^ laminarin determined a strong alkalinization of the extracellular medium followed by NADPH oxidase-dependent release of H_2_O_2_ triggering the subsequent induction of defense-related enzymes such as PAL, caffeic acid O-methyltransferase (CaOMT), and lipoxygenase. These enzymes are involved in phenylpropanoid, lignin and fatty acid biosynthesis, suggesting that laminarins mobilize the metabolic machinery of tobacco cells to elicit defense responses. The infiltration of laminarin restricted the progression of soft-rot disease caused by *P. carotovorum* in tobacco plants by inducing plant natural defense mechanisms [31]. Similarly, Aziz et al. [30] showed the elicitation of the early signalling events by laminarins in grape cells. In addition to the increased alkalization and higher release of H_2_O_2_ in the growth medium, laminarins also induced Ca^2+^ influx in grapevine cells. Cells grown in the medium supplemented with laminarins showed the activation of two mitogen-activated protein kinases, of defense-related genes and enzymes such as chitinase and β-1,3-glucanases, and increased biosynthesis of phytoalexins [30]. The foliar application of laminarins to the grapevine plants, conferred resistance against *B. cinerea* and *Plasmopara viticola*. However, laminarins did not induce hypersensitive response or cell death, but elicited the plants’ own innate immune system in both grapevine and tobacco [30,31]. Xin et al. [36] showed that laminarins from *L. digitata* protected tea plants (*Camellia sinensis*) against the tea green leafhopper (TLH), *Empoasca* (*Matsumurasca*) *onukii.* Laminarins were found to trigger immediate defense responses in plants by activating early defense-response genes, such as *MAPKs* and *WRKY,* and elevate the biosynthesis of SA and ABA, whilst also inducing oxidative bursts [36]. The induction of the MAPKs and WRKYs by laminarins regulates the activation of transcription factors, which subsequently modulate the activation of defense-responsive genes, such as *NPR1*, *PDF1*.2 [36]. The leaves of the tea plants sprayed with laminarins showed a higher activity of PAL, polyphenol peroxidase (PPO), chitinase, flavonol synthase and increased callose deposition. In addition to this, laminarin treatment increased tea volatile emissions involved in defense response. All these metabolic and defense responses helped laminarin-treated plants to reduce the TLH infestations [36]. All these results together suggest that laminarins from various seaweed extracts have the potential to induce early defense responses against different plant pathogens, including bacteria, fungi, and insects (Figure 1).

Menard et al. [32] showed that the sulphated form of β-1,3 glucan, β-1,3 glucan sulphate, is a more effective elicitor of plant defense responses than β-1,3 glucan. The comparative structural analyses of laminarins and sulphated laminarin (PS3) revealed that chain length was very important for the biological activity of both the compounds, and that the sulphation of the laminarin increased its bioactivity, which cannot be achieved by using other anionic groups [32]. In tobacco cells, the oxidative bursts induced by PS3 were Ca^2+^ dependent, but partially kinase independent. In contrast, laminarin induced oxidative bursts in a kinase-dependent manner only. The treatment of tobacco cells with PS3 determined electrolyte leakage, which caused the accumulation of SA and scopoletin, whereas such responses were not observed in the presence of laminarins [32,96]. Complementation assays in *A. thaliana* revealed that PS3 elicited the expression of ethylene- and SA-dependent PR proteins, while laminarins from *L. digitata* induced the expression of ethylene-dependent PR proteins only [32]. Laminarins are considered as a standard substrate for β-1,3 glucanases, whereas its chemical sulphation makes it resistant to degradation by β-1,3 glucanases [32]. The infiltration of PS3 into the leaves of transgenic PR1–β-glucuronidase (GUS) tobacco plants induced the expression of GUS, providing further evidence for the activation of the SA-dependent signalling pathway [33]. PS3 treatment of tobacco cells did not induce the expression of acidic PR1 proteins, which is considered a clear sign of SAR activation in plant cells [33,98]. The infiltration of tobacco leaves with 200 μg mL^−1^ of PS3 produced a 100% reduction in lesions upon TMV infection, while the same concentration of laminarins caused only a 60% reduction in lesions [33]. These results suggested that PS3 had stronger antiviral effects, as compared to laminarins, while the synergistic application of PS3 and laminarins together induced stronger oxidative bursts, as compared to laminarin and PS3 alone, but no synergy was observed in the expression of PR proteins [32,33]. These results provide an insight that the combination of the various forms of the bioelicitors can be further used for the development of potential novel biostimulant for plant disease management.

Unlike laminarins, PS3 did not elicit the early defense response, but induced resistance by enhancing prolonged plasma membrane depolarization in grapevine [28]. Microarray analysis revealed that PS3 induced 132 genes in grapevine, while laminarins from *L. digitata* induced only 94 genes. Interestingly, 94% of the induced genes were expressed in both the PS3 and laminarin treatments. Among the 33 genes expressed specifically in the PS3-primed grapevine leaves, most of the genes were involved in signalling pathways, such as phospholipase C, calmodulin, CBL-interacting protein kinase *(CIPK 14)*, serine/threonine-protein kinase (*AFC2*) and the transcription regulators *NAC78*, *AP2/ERF*, jumonji, squamosa-binding protein and ankyrin repeat 2 *(AKR2)*. PS3- and laminarin-treated plants showed the expression of the genes that are common in the SA- and JA- dependent pathways [28]. However, Trouvelot et al. [35] demonstrated that a foliar spray of PS3 induced a JA-dependent defense response in *Vitis vinifera* cv. Marselan against downy mildew caused by *P. viticola*. In addition to this, PS3 elicited NADPH oxidase-dependent oxidative burst and the expression of stilbene synthase *(STS)*, *PAL*, *LOX,* glutathione-S-transferase *(GST),* protease inhibitor *(PIN),* and basic class I chitinase *(CHIT1b)*. The treatment of PS3-primed leaves with 2-deoxy-d-glucose (i.e., an inhibitor of callose synthase) and 5, 8, 11, 14-eicosatetraynoic acid (i.e., an inhibitor of 9-lipoxygenase) reverted the PS3-induced resistance. This suggested that PS3 elicited the defense response by callose deposition and induced hypersensitive responses, such as cell death [35]. These results were further confirmed by Gauthier et al. [28] who demonstrated that PS3-priming induced the expression of the respiratory burst homolog gene (*RbohD*) which is involved in oxidative bursts and also *HSR203J* which is involved in HR responses in those grapevines leaves infected with *P. viticola* [28]. Adrian et al. [38] studied the effects of PS3 on the global metabolism of grapevine leaves infected with *P. viticola.* Erythritol phosphate, which is involved in the production of monoterpenes and sesquiterpenes, was found to be significantly induced in the leaves of grapevines treated with PS3, in response to *P. viticola* infection [38]. Figure 1 represents comparative molecular mechanisms involved in the induction of plant immunity by laminarins and sulphated laminarins. These results suggest that the laminarins, laminarin-derived products, and particularly sulphated laminarins, induced defense responses in treated plants by triggering various metabolic, physiological, and biochemical processes. 

### 2.4. Ulvans

Ulvans are sulphated polysaccharides, extracted from the cell wall of green seaweeds, in particular from various species of the genus *Ulva,* which generally accounts for 9–36% of their dry biomass [99,100,101]. Ulvans are extracted from the algal biomass either by acid or enzyme-based extraction procedures, and are mainly composed of sulphated rhamnose, uronic acids (glucuronic acid and iduronic acid) and xylose [100,102,103]. The structural backbone of ulvans consists of monosaccharides (e.g., rhamnose, xylose, glucuronic acid and iduronic acid) joined by α- and β-(1,4)-linkages with characteristic repeating disaccharide units (i.e., ulvano-biuronic acid, ulvano-bioses) [100,101]. Commercially, ulvans have been widely used as ingredients in food, pharmaceuticals, and biomedical applications. They possess immune-modulatory, anti-inflammatory, antioxidant, antibacterial, antiviral, anticoagulant, and antihyperlipidemic activities [100,101,104]. In recent years, ulvans have been used widely for the modulation of active defense mechanisms in plants against a broad range of pathogens [49,96,105]. Foliar spray applications of ulvans from *Ulva fasciata* on the leaves of bean plants significantly reduced the colonisation of epicotyl xylem vessels by *Fusarium oxysporum* and thereby reduced the development of *Fusarium* wilt [51]. Similarly, in apple leaves, the spraying of ulvans from *U. fasciata* was found to reduce the severity of *Glomerella* leaf spot (caused by *C. gloeosporioides*) by 50%, by inhibiting the formation of appressoria [105]. Likewise, ulvans from *U. lactuca* and oligo-ulvans, prepared by enzymatic degradation, triggered a rapid and transient oxidative burst, and elicited the antioxidant activity of related defense-responsive enzymes, thereby inducing resistance in the apple fruits against *Penicillium expansum* and *B. cinerea* infections [106]. A commercial biostimulant based on ulvans (from hydrolysis of *Ulva armoricana*) (Elicityl Ltd., Crolles, France) was found to confer resistance against *C. gloeosporioides,* causing anthracnose disease in papaya. The induced resistance was attributed to an increased activity of defense-related enzymes involved in antioxidant metabolism [55]. Delgado et al. [54] demonstrated that the foliar application of ulvans induced resistance in bean plants against rust, caused by *Uromyces appendiculatus* and angular leaf spot, caused by *Pseudocercospora griseola*. 

Unlike laminarins, ulvans induced resistance against *Alternaria brassicicola* and *Colletotrichum higginsianum*, independent of their degree of sulphation [58]. Briand et al. [105] showed that a foliar spray of ulvans, prepared from *U. armoricana*, induced the expression of genes involved mainly in the defense response in *Medicago truncatula*. These applications conferred significant protection for *M. truncatula*, pea, and pepper against *C. trifolii*, *Mycosphaerella pinodes* and *Phytophthora capsicum,* respectively [105]. Similarly, a foliar spray of ulvans on the leaves of the common bean (*Phaseolus vulgaris*) conferred resistance against anthracnose disease, caused by *Colletotrichum lindemuthianum* by inducing the activity of defense-related enzymes [57]. In a different study, Ben Salah et al. [47] showed that a twig of olive (*Olea europaea*) dipped in a solution of ulvans (2 g/L) stimulated phenolic metabolism, and thereby increased its resistance to the *Verticillium* wilt of olive (VWO), caused by *V. dahliae*. The priming of rice and barley by ulvans elicited defense responses in the whole plants and reduced the incidence of powdery mildew caused by *Blumeria graminis*, by 45% in wheat and by 80% in barley, respectively [52]. It was found that wheat and rice cells primed with ulvans did not induce oxidative bursts independently, but rather induced oxidative bursts in the cells which already had been primed with chitin and chitosan [52]. de Freitas and Stadnik [58] showed that a foliar spray application of ulvans from *U. fasciata* reduced the severity of colonisation by *A. brassicicola* of wild-type (WT) *A. thaliana*, but did not protect *AtrbohD* plants impaired in NADPH oxidase, which is required for the production of ROS during oxidative burst. Furthermore, ulvan priming reduced electrolyte loss by 130% in WT, but failed to control it in the *AtrbohD* mutant. The infiltration of diphenyleneiodonium into the leaves of WT and *AtrbohD* impaired NADPH oxidase activity and hydrogen peroxide accumulation, thus reverting the ulvan-induced resistance in *A. thaliana* against *A. brassicicola* [58]. Taken together, ulvans induced disease resistance in *A. thaliana* by stimulating NADPH oxidase-dependent ROS accumulation but did not activate the hypersensitive response. Ulvans failed to induce the defense response in *AtrbohD*, as the treatment lacked the respiratory burst oxidase homologues, required for triggering of cell-to-cell signalling cascades which result in ROS production [58]. Ulvans and methyl jasmonate (MeJA) were found to induce similar types of responses in *M. truncatula* [54]. Microarray analysis revealed a 40% identical expression in the plants treated with MeJA and ulvans, and these genes were related to JA-dependent defense responses, including lipoxygenase, hydroxyproline-rich glycoproteins, proline-rich proteins, cysteine-rich antifungal proteins (i.e., defensin) and wound-induced proteins [54]. Treatments with ulvans modulated the expression of genes involved in phytohormone metabolism. Gibberellic Acid Insensitive (GAI), Repressor of GAI (RAG) and Scarecrow (SCR) (GRAS) transcription factors (TF) are expressed in response to gibberellins and bacterial and fungal elicitors. Ulvans induced the expression of GRAS TFs, suggesting that ulvan treatments were able to regulate the crosstalk occurring between elicitors and gibberellin responses [54,107,108]. Jaulneau et al. [54] demonstrated that a foliar spray of ulvans on the leaves of *A. thaliana* induced the expression of *PDF1.2*, which was significantly reduced in ulvan-sprayed jasmonic acid resistant, *jar1.1* and the abscisic acid deficient, *aba3.1* mutants. On the contrary, the SA-dependent genes *PR1a* and *PR5* were not induced by the treatment of ulvans extracted from *U. armoricana*, suggesting that the activated JA-dependent defense-responsive pathway acted synergistically with the ABA pathway. Collectively, these results suggested that, in general, ulvans induced resistance in a JA-dependent fashion, thereby regulating cross-talk amongst various defense-responsive processes.

In addition to these bioactive polysaccharides, oligofucans isolated from *Pelvetia canaliculata* elicited a defense response in tobacco suspension culture by inducing alkalization and oxidative bursts in treated cells [59]. The tobacco cells treated with oligofucans exhibited the strong induction of PAL, LOX, PR proteins, and phytoalexin [59]. The infiltration of oligofucans in the leaves of tobacco, systemically induce the SA-dependent defense response against TMV [59]. The foliar spray of tomato seedlings with oligoulvans and oligoglucuronans from *U. lactuca* showed a strong induction of PAL activity, followed by the accumulation of SA and phenolic compounds [60]. The oligoulvan- and oligoglucuronan-treated seedlings showed reduced susceptibility towards wilt disease caused by *F. oxysporum* f. sp. *lycopersici*.

### 2.5. Phenolics 

Polyphenols represent a class of secondary metabolites that are widely distributed in various seaweeds [109]. Polyphenolic compounds derived from marine sources are widely used as antibacterial and antiviral agents in pharmaceuticals [110,111,112]. Phlorotannins are unique phenols (tannins) found in brown algae. Structurally, phlorotannins consist of monomers of phloroglucinol (1,3,5-trihydroxybenzene) joined by either ether (C−O) or aryl−aryl (C−C) linkages [112,113]. These unique polyphenols are known to play the role of inducing chemical defenses against herbivory [114]. The bioactive phenolic compounds extracted from *Sargassum muticum* and *Ascophyllum nodosum* posses radical scavenging activities [115,116]. Jormalaninen et al. [117] showed that the accumulation of phlorotannins was induced in *Fucus vesiculosus* in the presence of two species of snails, i.e., *Theodoxus fluviatilis* and *Physa fontinalis*, which are known to feed on brown algae. Arnold et al. [118], proposed that phlorotannins were synthesized as reactive, polyphenolic secondary metabolites in specialised cells called physodes (i.e., intracellular vesicular inclusions) of brown algae, and then converted to unreactive forms as cell wall components. Thus, an increased accumulation rate of phlorotannins, upon attack by herbivores, is not necessarily the result of chemical defense mechanisms; they are likely required for the reconstruction of the damaged cell walls. Polyphenolics and phlorotannins extracted from *F. vesiculosus* by ultrasound-assisted extraction exhibited antioxidant properties [119]. Amongst the brown algae, *A. nodosum* is a rich source of phlorotannins, and an extraction of these was found to reduce the occurrence of food-borne pathogens in pigs, without impacting the normal physiology of the intestine [112,120]. Eckol, a phlorotannin obtained from *Ecklonia maxima,* was shown to stimulate plant growth and seed germination [121]. Foliar applications of eckol induced myrosinase activity in treated cabbage and stimulated natural defense responses related to aphid infestation by regulating the glucosinolate-myrosinase system [62]. The use of phenolics as constituents of biostimulant formulations for plant disease management needs to be further explored in much more detail. Taking into consideration their antioxidant and antiviral properties, demonstrated in both plants and in other experimental models (i.e., animals) [112,119], these bioactive compounds derived from seaweeds are likely to be an important resource for improving natural defense mechanisms in these forms of life, leading to the development of novel commercial products of benefit to plants, animals, and microbes.

## 3. Seaweeds: Sources of Extracts Used as Biostimulants

The presence of bioactive compounds has fuelled the increasing interest in utilising the seaweeds as a sustainable raw material [119]. The biostimulants prepared from the various seaweeds elicits plant defense by following a unique mode of action (Table 2). 

### 3.1. Ascophyllum Nodosum

The phaeophycean rockweed, *Ascophyllum nodosum,* is a widely distributed intertidal species that can form dense, mono-specific, sub-marine forests around the periphery of the North Atlantic Ocean [91]. Rockweed is commonly found on the north-western coast of Europe, east Greenland, and the North East coast of North America. Similar to other intertidal species, *A. nodosum* is able to survive extreme climatic challenges [13], and the brown alga is a rich source of secondary metabolites [122,123]. *A. nodosum* is also a significant source of sterols, phlorotannins, fucoidans, ascophyllan, mannitol, alginic acid and laminarin [20,123,124,125,126,127,128]. These unique characteristics make *A. nodosum* one of the most important sources of raw materials for the commercial production of bioactive compounds [13,20,129]. The elicitors present in the different extracts and formulations were shown to reduce disease severity and incidence, i.e., ameliorate biotic stresses in plants such as tomato, strawberry, carrot, cucumber and the model plant *A. thaliana* (Table 2) [20,130,131,132,133,134]. 

A liquid extract prepared from *A. nodosum* was found to elicit D-glycanases activities [135]. Betaines present in an alkaline extract of *A. nodosum* reduced the replication of root-knot nematode, *Meloidogyne javanica*, in mono-xenic cultures of *A. thaliana* [136]. Alternate applications of Stimplex^®^ (Acadian Seaplants Ltd., Dartmouth, NS, Canada), an Environmental Protection Agency (EPA)-registered pesticide, in combination with the fungicide chlorothalonil (2 g L^−1^) reduced the progression of *Alternaria cucumerina*, *B. cinerea, Didymella applanata* and *F. oxysporum* in cucumber plants grown under greenhouse conditions [137]. Similarly, the integrated use of an alkaline extract prepared from *A. nodosum* (ANE) with fungicides (i.e., chlorothalonil and cupraneb) reduced the severity of an infection caused by *A. solani* and *Xanthomonas campestris* pv. *vesicatoria* in tomato and sweet pepper, under greenhouse and field conditions [130,138]. The increased induced resistance to the pathogens observed was likely due to the enhanced activity of PAL, PPO, peroxidase (PO), and β-1,3 glucanases [130]. 

Tomato and sweet pepper plants treated with ANE showed a significantly higher transcript expression of *PIN II* (proteinase inhibitor) and *ETR-1* (ethylene receptor) genes, which are involved in JA and ET-mediated defense signalling pathways. Likewise, no significant changes were observed in the expression of the *PR-1a* in both crops [138]. In addition to the defense-responsive genes, ANE-treated tomato and sweet pepper plants showed a higher expression of isopentenyltransferases *(IPT)*, Indole acetic acid (*IAA*) and *Ga2Ox*, genes that are known to be involved in hormonal biosynthesis [138]. Taken together, these results suggest that the *A. nodosum* extract induced innate immunity by regulating crosstalk between defense-responsive signalling pathways and hormonal biosynthesis. 

A foliar spray of 0.2% ANE improved resistance against *Podosphaera aphanis*, which is the causative agent of powdery mildew in strawberries by enhancing the biosynthesis of secondary metabolites and defense-related enzymes [131]. Similarly, a foliar spray of 0.2% of Stimplex^®^ controlled the progression of fungal pathogens, i.e., *Alternaria radicina* and *B. cinerea* in carrot by inducing the activity of several defense-related enzymes, such as PO, PPO, PAL, chitinase and β-1,3-glucanase [133]. The treated carrot plants showed a higher expression of *PR-1*, chitinase, lipid transfer protein *(LTP*), *PAL*, *CHS*, *NPR-1* and *PR-5* in response to the pathogen [133]. 

Marmarine^®^ (International Ferti Techology Corporation, Amman, Jordan), induced defense responses against *Phytophthora melonis* in cucumber [139]. The combination of foliar and root drench of Marmarine^®^ was found more effective in inducing disease resistance, as compared to foliar and root drench alone. Marmarine^®^ induced systemic resistance in cucumber by increasing the expression of cucumber pathogen-induced 4 (*Cupi4*), lipoxygenase (*LOX*), *PAL*, and galactinol synthase (*GolS*) genes involved in defense responses [139].

*A. nodosum* extracts prepared by Acadian Seaplants Limited, induced systemic defense responses against *Pseudomonas syringae* and *S. sclerotiorum* by inducing the expression of the genes involved in the JA-dependent pathway [145]. The pre-treatment of Stella Maris^®^ (Acadian Seaplants), a commercial extract prepared from *A. nodosum,* inhibited the growth of several bacterial pathogens in *A. thaliana* by inducing a strong oxidative burst of reactive oxygen species (ROS) [132]. In addition to this, Stella Maris^®^, also induced the expression of cytochrome P450 family polypeptide (*CYP71A1*2), an antimicrobial phytoalexin known to damage the cell wall and disrupt the metabolism of bacterial pathogens [132]. The expression of other defense-related genes, such as *WRKY30* and *PR1*, was also find higher in *A. thaliana* in response to a *P. syringae* infection [132]. 

The combination of liquid seaweed extract prepared from *A. nodosum* (Acadian Seaplants) and chitosan reduces the severity of *Fusarium*-head-blight (FHB) caused by *Fusarium graminearum* by eliciting the expression of pathogenesis-related genes (*TaPR1.1, TaPR2, TaPR3, TaGlu2*) and defense-related enzymes [150]. *F. graminearum* infection results in the loss of yield and accumulation of mycotoxin in the infected grain, causing reduced quality of grains for animal and human consumption [171]. The levels of mycotoxins, deoxynivalenol and sambucinol, were found to be lower in those wheat grains harvested from the plants treated with a combination of liquid seaweed extract and chitosan [150]. Similarly, the combination of liquid seaweed extract prepared from *A. nodosum* and chitosan inhibited the growth of *Erisyphe pisi* causing powdery mildew in pea [172]. Seasol Commercial^®^ (Seasol International, Bayswater, Australia), is an alkaline hydrolysis product prepared from *Duvillaea potatorum* and *A. nodosum* and was found to reduce the progression of *Plasmodiophora brassicae*, causing club-root in broccoli [144]. The treatment of the broccoli with the seaweed extract reduced the number of the plasmodia formed in the roots [144]. Islam et al. [134] studied the protective effects of Seasol^®^, ANE and an alkaline extract of *Duvillaea potatorum* on *A. thaliana*, before and after inoculation with the root pathogen *Phytophthora cinnamomi,* using a transcriptomics approach. Global transcriptomics analysis revealed that Seasol Commercial^®^ reduced the progression of *P. cinnamomi* by regulating proteolytic pathways, respiratory burst, and various defense-related responses. Overall, each seaweed extract acted differently, but all were successful in inducing plant defense-related genes [134]. The root drench treatment of 0.5% Dalgin^®^ (Sustainable Agro Solutions, Lleida, Spain), is another commercial extract prepared from *A. nodosum* that can significantly reduce the severity of a *P. capsici* infection of tomatoes by inducing the expression of several defense-related genes and also the activity of many oxidative enzymes [142]. Another report published by Somai-Jemmali et al. [141] showed that Dalgin Active^®^, consisting of 22.6% *A. nodosum* extract, 0.135 % vitamins, 1.43% nitrogen and 6.78% free amino acid, induced defense responses in both bread and durum wheat against the fungal pathogen *Z. tritici.* A foliar spray of this extract induced the plant defense response by eliciting expression of multiple genes involved in defense responses, antioxidant metabolism and both the phenylpropanoid and octadecanoid pathways [141]. 

The extensive cultivation of the seaweed *Kappaphycus alvarezii* (Rhodophyta)*,* a commercially important carrageenophyte, is hampered by infestations by the endo-epiphytic red alga *Neosiphonia apiculata* [147]. An extract with the abbreviated name AMPEP (*Ascophyllum* Marine Plant Extract Powder) [173,174], prepared from the soluble powder extract of *A. nodosum*, exhibited a “vaccine-like” effect on *K. alvarezii*, as tested in Brazil, and induced the natural defense in this alga by reducing the effects of surface level oxidative bursts. AMPEP also increased the daily growth rate and carrageenan yield and quality of the treated crops [147]. AMPEP, as a biostimulant solution for promoting the growth of *K. alvarezii* propagules was found to reduce the percentage occurrence of *Neosiphonia* [148], and improved carrageenan quality [149] in studies conducted in SE Asia.

### 3.2. Ecklonia Maxima

*Ecklonia maxima*, commonly known as kelp or “sea bamboo”, is a brown seaweed (Phaeophyceae) distributed mainly in the southern hemisphere, on the southern Atlantic coast of Africa [175]. *E. maxima* is known to be a rich source of polyamines, phlorotannins, and of ACC (1-amino-cyclopropane-1-carboxylic acid) [176,177,178]. The biomass of this brown seaweed has been largely used as an integral constituent of animal feed, nutritional supplements, and soil conditioner [177]. Kelpak^®^ is an extract of *E. maxima* (Kelp Products International, Simon’s Town, South Africa) which is prepared by using a cold, cell-burst technique [179]. Kelpak^®^ contains various growth-promoting compounds and has been reported to improve plant productivity under stress conditions [176,180,181,182]. An application of the liquid extract reduced occurrence of *Verticillium* wilt in green pepper caused by *Verticillium dahliae* [151]. A soil drench treatment of tomato with Kelpak^®^ significantly reduced the infestation of the root-knot-causing nematode *Meloidogyne incognita*, whilst foliar applications of Kelpak^®^ were not found to be effective in controlling *M. incognita* infection [152]. The commercial extract prepared from *E. maxima* adversely affected the hatching and sensory perception of root-nematodes in vitro. This suggested beneficial applications in controlling the infection of tomato roots by *M. chitwoodi* and *M. hapla* [153]. 

### 3.3. Sargassum spp.

*Sargassum* is one of the largest genera of brown algae. Due to its abundance, it is an integral part of several marine ecosystems; however, some species are invasive in nature [183]. For example, *Sargassum muticum*, commonly known as “Japanese wireweed”, a native to Japan, has invaded wide areas of the Atlantic coasts of Europe since its introduction in the region, and now it is one of the most abundant Sargassaceae species used on the European markets [184]. *S. muticum* offer an auspicious source of bioactive compounds as they develop chemical defense mechanisms which helps the invasive seaweed to establish in new, highly dispersed geographical environments [184]. A laboratory-scale, aqueous extract prepared from *S. muticum* represents a natural source of bioactive molecules, such as phlorotannins, laminarins, alginic acid, phenolic compounds, antioxidants, carotenoids, and anticancer compounds such as fucoxanthin [185]. *Sargassum fusiforme*¸ also known as jade grass, is a perennial, warm-temperature alga, mainly found in south coastal areas of China, Japan, North Korea and South Korea [186]. The bioactive compounds present in an aqueous extract of *S. fusiforme* induced resistance in *Solanum lycopersicum* against several pathogens [167]. Foliar applications of *S. fusiforme* extract on tomato plants reduced disease severity caused by powdery mildew by 37%, while the severity of late blight, caused by *Phytopthtora infestans*, and of gray mold, caused by *B. cinerea*, was reduced by 36 and 80%, respectively. The extract did not exhibit any direct antifungal activity, but rather induced systemic defense responses in tomato against pathogen infestations [167]. On the contrary, the organic solvent and aqueous extracts from *S. vulgare* inhibited the mycelial growth of *F. oxysporum* f. sp. *tuberosi* and controlled the progression of *Fusarium* dry rot on those potato tubers treated with the extracts [165,187]. Similarly, Ammar et al. [164] demonstrated that an aqueous extract from *S. vulgare*-controlled *Pythium* leek disease caused by *P. aphanidermatum*. Ali et al. [168] showed that the foliar application of an extract prepared from *S. vulgare* resulted in a significant reduction in the progression of disease caused by *X. campestris* and *A. solani* in tomato and sweet peppers under both, greenhouse and field conditions. *S. vulgare* extract induced resistance in treated plants by regulating expression of genes involved in defense-signalling pathways (i.e., *PR-1a*, *PINII*, and *ETR-1*) and phytohormone biosynthesis [168]. The foliar application of an extract prepared by the alkali treatment of *Sargassum polycystum* was reported to reduce leaf fall disease caused by *Phytophthora palmivora* by inducing systemic acquired resistance. The application of the extract induced enzymatic activities of catalase, endo-1,3-d-glucanase, and peroxidase in response to *P. palmivora* [166]. 

In a recent study, the foliar treatment of laboratory-scale extracts (i.e., hot and cold water and alkali) from *Sargassum tenerrimum*, at both vegetative and reproductive stages, reduced the progression of charcoal-rot caused by *Macrophomina phaseolina* [163]. Endogenous phytohormone analysis of the treated plants revealed higher salicylic acid (SA) accumulation in the treated vs. control groups, suggesting that induction of SA-dependent, defense pathways against *M. phaseolina* was responsible for the observed, reduced infection [163]. The cross talk of endogenously produced phytohormones, in response to different stresses, is an energy-efficient strategy of plants [188]. A higher accumulation of abscisic acid (ABA) was reported to increase the susceptibility of plants to pathogen infection [189]. The extract prepared from *S. tenerrimum* significantly reduced ABA accumulation in tomato plants infected with *M. phaseolina*. In addition, this *Sargassum* extract also induced endogenous phytohormones such as indoleacetic acid (IAA) and kinetin in *M*. *phaseolina*-infected tomato plants, during the vegetative and reproductive stages of pathogen development [163]. Taken together, these results suggest that bioactive formulations prepared from various *Sargassum* spp. may elicit the innate defense responses of plants by regulating the crosstalk between endogenous phytohormones and other biological processes. 

### 3.4. Kappaphycus Alvarezii

Elkhorn sea moss, *Kappaphycus alvarezii* (Rhodophyta), is an industrially important red seaweed, primarily cultivated for the extraction of the phycocolloid carrageenan and some quaternary ammonium compounds [190,191]. K-sap is a commercial name given to a product obtained after mechanical crushing of the fresh seaweed. It contains phytohormones, such as IAA, kinetin, trans-zeatin and gibberellic acid (GA3) [154,192,193]. The foliar application of 5% of K-sap induced systemic defense responses in tomato plants against *M. phaseolina* [154]. The expression of the defense-response gene was found to be higher in the K-sap-treated tomato plants. The application of K-sap to *M. phaseolina*-inoculated plants induced the expression of SA-dependent *PR-1*, *PR-3* coding chitinase and *PR-5* coding osmotins by 2-fold, 4.5-fold, and 1194-fold, respectively. The trend in gene expression was supported by higher levels of SA accumulation [154]. K-sap was found to modulate endogenous plant phytohormone biosynthesis in response to *M. phaseolina* infections, thereby mediating crosstalk between different signalling pathways [154]. The potential for bioactive compounds from *K. alvarezii* needs to be explored further in priming plant defense against plant pathogens.

### 3.5. Gracilaria spp.

The genus *Gracilaria* belongs to the Rhodophyta and its members are distributed throughout the world in tropical and temperate waters [194,195]. Most of the species are commercially exploited primarily for their use as raw materials in agar production [194]. However, there have been a few studies focusing on the use of *Gracilaria* extracts as plant biostimulants [196,197,198,199,200]. *Gracilaria* extracts are rich sources of fatty acids, florisides, sterols, polyols, terpenoids and hydrocolloid polysachhrides [200]. Palmitic acid and the agarans isolated from the aqueous extract of *Gracilaria caudata* and *G. domingensis* promoted the early growth of lettuce [200]. Soliman et al. [156] showed that organic fractions of aqueous extract obtained from *G. confervoides* had antifungal activities against the plant pathogens *Rhizoctonia solani*, *Fusarium solani* and *M. phaseolina*. The maximum reduction in the radial growth of the pathogen on potato dextrose agar was observed when the medium was amended with a chloroform fraction of the aqueous extract of *G. confervoides*. The incidence of the disease caused by these pathogens was reduced when cucumber plants were grown in the soil amended with the powder of *G. confervoides* [156]. Silver nanoparticles synthesized using *G. cortica* exhibit antifungal activity against *Candida albicans* and *C. glabrata* [201].

### 3.6. Ulva spp.

The genus *Ulva*, commonly known as “sea lettuce”, is one of the most abundant green macroalgae (Chlorophyta) throughout the world [102]. *Ulva* spp. are perhaps the best researched members of the Ulvophyceae and indeed some species are used as experimental models in order to study macroalgal development, growth, and morphogenesis. *Ulva* biomass is used in the restoration of degraded environments [202,203]. Sea lettuce is a rich source of unique, sulphated polysaccharides called ulvans. These contain rhamnose, sulphate, xylose, iduronic acid, galactose, and glucose [54,204], and biomass may also used as a source of biofuel [205]. Cluzet et al. [160] demonstrated that the elicitors present in an extract of *Ulva* sp. prepared by hot water extraction induced immunity in *Medicago truncatula* against *Phytophthora parasitica* var. *nicotianae* by triggering the induction of *PR-10*, the hallmark of a plant defense response against pathogens. In addition to this, the foliar spray of an extract from *Ulva* sp. also protected *M. truncatula* from the fungal pathogen *Colletotrichum trifolii*, which is the causative agent of anthracnose. The differential expression analysis of expressed sequence tags (ESTs), obtained by a microarray analysis, revealed the induction of 152 genes in *M. truncatula* sprayed with the extract from *Ulva* sp. These ESTs were mainly involved in the biosynthesis of phytoalexins, pathogenesis-related proteins and cell wall proteins [160]. The bioactive compounds present in the ethyl acetate fraction derived from an aqueous extract of *U. lactuca* reduced the post-harvest losses in citrus by controlling the occurrence of green mould caused by *Pencillium digitatum* [159]. The elicitors isolated from *U. lactuca* stimulated the natural defense in the treated tomato seedlings against *F. oxysporum* f. sp. *lycopersici* [60]. A polysaccharide-enriched extract from *U. lactuca* showed a significant reduction in the progression of *A. solani* infection in tomato seedlings, as compared to extracts obtained from *Caulerpa sertularioides*, *Padina gymnospora* and *Sargassum liebmanni* [157]. A foliar spray of 0.1 mg mL^−1^ of the *U. lactuca* extract on tomato seedlings showed highest induction of activities of defense-related proteins, such as PPO, POD, and trypsin inhibitor [157]. The application of *U. lactuca* extract induced the expression of genes involved in wound response, JA biosynthesis, and defense-response. Interestingly, *PAL* was downregulated in those tomato plants treated with *U. lactuca* [157]. Heliosol^®^ (Action-Pin, France), a commercial extract derived from the extract of *Ulva armoricana*, induced natural defenses in bean, grapevine and cucumber against powdery mildews caused by *Erysiphe polygoni*, *E. necator* and *Sphareotheca fuliginea*, respectively [154]. In the same study, Jaulneau et al. [154,155] showed that the infiltration of the extract of *U. armoricana* into the leaves of tobacco, expressing the reporter construct P1Lox: GUS, was a determinant for a higher β-glucuronidase activity. The aqueous and methanolic extracts from *U. fasciata* reduced both mycelial growth and the conidial germination of *Colletotrichum lindemuthianum*. Foliar applications of these extracts on to the leaves of Phaseolus vulgaris induced defense mechanisms against the anthracnose disease caused by *C. lindemuthianum* [53]. In another study, Rajesh et al. [206] synthesized silver nanoparticles impregnated with an ethyl acetate fraction of *U. fasciata*, they reported a reduced growth of *X. campestris*. These results indicate that *Ulva* spp. have an immense potential for being used in various disease management programs; however, current research lacks more in-depth knowledge of the bioactive compounds present in *Ulva* spp. and their modes of action.

In addition to the well-characterised seaweeds, others have also been reported for being used as a source of bioactive compounds for the management of plant diseases. The mixture of the alcoholic and aqueous extract from the tropical brown alga, *Turbinaria conoides*, demonstrated an antifungal property against the root rot pathogen *F. oxysporum* [169]. Esserti et al. [170] showed that methanolic extracts from the brown seaweeds, *Cystoseira myriophylloides*, *L. digitata*, and *F. spiralis*, controlled the progression of disease caused by *V. dahliae* and *Agrobacterium tumefaciens* in tomato plants. The treated plants showed reduced symptoms of disease, not by inhibiting pathogen growth, but by inducing plant defense responses, which included the enhanced synthesis of defense-responsive enzymes, such as polyphenol oxidase and POD [170]. An extract from *A. spicifera* conferred resistance against *X. campestris* and *A. solani* in tomato and sweet pepper by modulating the expression of genes involved in defense signalling and phytohormone biosynthesis [168]. Similarly, Ramkissoon et al. [158] determined the elicitor activity of tropical seaweeds, *U. lactuca*, *S. filipendula* and *Gelidium serrulatum*, luxuriantly found along the coast of Trinidad and the southern Caribbean, against *X. campestris* and *A. solani* infection in tomato. The alkaline extract prepared from *U. lactuca* and *S. filipendula* conferred resistance against *X. campestris* and *A. solani* infection in tomato plants by inducing the JA-dependent signalling system, while the alkaline extract from *G. serrulatum* induced both SA- and JA-dependent signalling pathways against both pathogens. 

## 4. Conclusions

Climate change and urbanisation have undoubtedly increased the pressure on the agricultural sector to improve the productivity and quality of all staple crops. Rapid changes in environmental conditions have detrimental effects on plant immunity and their ability to fight against pathogens, thus augmenting the pressure on the agricultural sector to increase the productivity of crops [207]. The expanded, continuous use of synthetic chemicals to reduce the growth of pathogens is no longer a viable option because of their deleterious effects on human health and the environment, thus affecting short- and long-term sustainability. 

**Figure 2:**
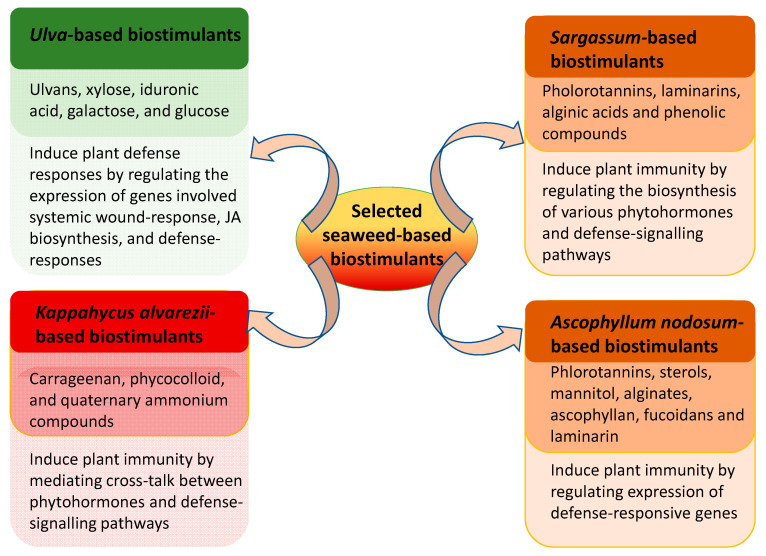
Bioactive components present in various seaweed extracts and plausible defense mechanism elicited by these extracts that induce plant immunity against pathogens.

To the rescue? Seaweed-based bio-elicitors offer a sustainable alternative for enhancing plant immunity against pathogens. Particularly during the last decade, the booming biostimulant industry has been characterized by the increasing utilisation of various seaweed-based extracts, in particular for relieving abiotic stresses. There is growing consensus that their applications have contributed to the reduction in synthetic chemical inputs to the environment [20]. Although the increasing number of reports on the use of seaweed extracts and their various, constituent bioactive compounds is encouraging, it is important to have a better understanding of their holistic modes of action and biotic responses, particularly in improving plant immunity (Figure 2). The biostimulant activity of various formulations seems to be strongly influenced by the extraction procedure, and so too the provenance/origin of the raw materials, seasonality (particularly in those originating in temperate areas), and ultimately in the potential synergistic/antagonistic activities of the numerous constituents of the extracts. 

In the current scenario, there is a requirement for a thorough re-evaluation of many biostimulants that have been revealed as promising in laboratory settings, and the development of products which are useful and commercially viable in real-world conditions.

The information summarized in this review aimed at providing a platform to researchers, emphasizing the necessity to evaluate thoroughly modes of actions in seaweed extract products in inducing plant immunity; it was also intended to generate a plethora of research questions that need to be answered, such as: will the combination of different seaweed extracts have synergistic effects and increased beneficial bioactivity? Is the usage of various bioactive compounds isolated from crude extracts commercially viable? Should the research focus more on the usage of seaweeds as enhancers of plant growth/yield, or as potential pest control agents? Do the modes of applications (i.e., drench or spray) and frequency of applications have different metabolic responses? Clearly, further research aiming at the identification, isolation and characterization of the bioactive compounds present in the extracts of various seaweeds will elucidate some of these important aspects. 

## Figures and Tables

**Table 1:** Seaweed-derived elicitors (phyco-elicitors) and their roles in the defense mechanism of plants.

Elicitors	Source	Mode of Application	Mode of Action	Reference
Sodium alginate	Commercial ^†^	Foliar spray	Elicited resistance in tomato against *Alternaria solani* by regulating expression of defense responsive genes and antioxidative enzymes	[24]
Alginate	*Fucus spiralis, Bifurcaria bifurcata*	Root soaking	Stimulation of natural defense of the roots of date palm	[25]
Alginate-derived oligosaccharides	Kelp	Cotyledon assay	Stimulated the accumulation of phytoalexin and phenylalanine ammonia lyase (PAL) in soybean cotyledon	[26]
Alginate oligosaccharides	Source unknown	Root drench	Elicited disease resistance against *Pseudomonas syringae* by inducing salicylic acid (SA)-defense pathway	[27]
Laminarin	*Laminaria digitata*	Foliar spray	Primed grapevine against *Plasmopara viticola* by inducing SA and ROS-dependent pathways	[28]
Laminarin	*L. digitata*	Foliar spray	Reduced *Botrytis cinerea, Sphaerotheca macularis and Mycosphaerella fragariae* infection in strawberry	[29]
Laminarin	*L. digitata*	Foliar spray	Increased protection of grapevine against *B. cinerea* and *P. viticola* by inducing expression of defense-related gene, accumulation of phytoalexins, chitinase, and β-1,3-glucanase activities	[30]
Laminarin	*L. digitata*	Leaf infiltration	Strong reduction in soft rot disease caused by *Erwinia carotovora* in tobacco	[31]
Laminarin	*L. digitata*	Leaf infiltration	Induced SA dependent defense signalling pathway in *Arabidopsis* and tobacco	[32]
Laminarin	*L. digitata*	Leaf infiltration	Elicited defense response against tobacco mosaic virus (TMV) in tobacco by regulating the expression of genes involved in phenylpropanoid pathway	[33]
Laminarin	*L. digitata*	Foliar spray	Reduced infection of powdery mildew in grapes	[34]
Laminarin	*L. digitata*	Foliar spray	Induced defense response in *Vitis vinifera* against *P. viticola* by regulating hypersensitive response and expression of defense-responsive genes	[35]
Laminarin	*L. digitata*	Foliar spray	Elicited defense responses in tea against the piercing herbivore *Empoasca (Matsumurasca) onukii*	[36]
Laminarin	*L. digitata*	Foliar spray	Increased elicitation of defense response against downy mildew in grapevine	[37]
Laminarin	*L. digitata*	Leaf disc assay	Elicited defense responses in leaves of grapevine against *P. viticola*	[38]
Iodus 40 (Laminarin)	*L. digitata*	Foliar spray	Improved defense response in wheat against powdery mildew infection	[39]
λ-carrageenan	Commercial ^†^	Foliar spray	Suppressed Tomato Chlorotic Dwarf Viroid replication by inducing the expression of the jasmonic acid (JA)-responsive gene	[40]
Ɩ-carrageenan	Commercial ^†^	Foliar spray	Induced defense response against *Trichoplusia ni* by modulating glucosinolate metabolism and expression of defense-responsive gene	[41]
λ-carrageenan	Commercial ^†^	Foliar spray	Elicited defense response in *Arabidopsis* against *Sclerotinia scleortiorum* by SA-independent defense signalling pathway	[42]
λ-carrageenan	*Acanthophora spicifera*	Foliar spray	Elicited *Hevea brasiliensis* defense against *Phytophthora* *palmivora* by inducing the SA-dependent defense signalling pathway	[43]
λ-carrageenan	*Gigartina acicularis, Gigartina pistillata*	Leaf infiltration	Induced resistance in *Nicotiana tabacum* against *Phytophthora parasitica* by regulating the expression of defense-related genes	[44]
λ-carrageenan	Commercial ^†^	Foliar spray	Elicited SA- and JA- dependent signalling pathways in wheat against Septoria tritici blotch caused by *Zymoseptoria tritici*	[45]
κ-carrageenan	*Kappaphycus alvarezii*	Foliar spray	Reduced anthracnose disease caused by *Colletotrichum gloeosporioides* in *Capsicum annuum* by inducing the defense-related antioxidant enzyme peroxidase	[46]
κ-carrageenan	*Hypnea musciformis*	Leaf infiltration	Activated SA- and jasmonic acid/ethylene (JA/ET)- defense signalling pathways and confers resistance against TMV	[47]
Oligo-carrageenan	*TMV, B. cinerea Pectobacterium carotovorum*	Foliar spray	Reduced progression of pathogen in tobacco plants by inducing synthesis of secondary metabolites	[48]
Alginate, carrageenan, laminarin Ulvan	Commercial ^†^ *Ulva lactuca*	In vitro assay	Ulvan and alginates reduced verticillium wilt of *Olea europaea* caused by *Verticillium dahliae* by stimulating phenolic metabolism	[49]
Ulvan	*U. lactuca*	Foliar spray	Elicited defense response against *Alternaria brassicicola* and *Colletotrichum higginsianum*	[50]
Ulvan	*U. lactuca*	Foliar spray	Controlled *Fusarium* wilt in *Phaseolus vulgaris* caused by *Fusarium oxysporum*	[51]
Ulvan	*Ulva fasciata*	Foliar spray	Elicited resistance against powdery mildew in wheat and barley	[52]
Ulvan	*U. fasciata*	Foliar spray	Induced resistance in *P. vulgaris* against anthracnose disease caused by *Colletotrichum lindemuthianum*	[53]
Ulvan	*Ulva armoricana*	Leaf infiltration, Foliar spray	Activated plant immunity through JA-signalling pathway	[54]
Ulvan	*Ulva* sp.	In vitro assay	Reduced Anthracnose disease caused by *C. gloeosporioides* in papaya by inducing antioxidant defense enzyme activity	[55]
Ulvan	*U. fasciata*	Foliar spray	Elicited the defense in *P. vulgaris* against bean rust and angular leaf spot	[56]
Ulvan	*U. fasciata*	Foliar spray	Increased defense responses in *P. vulgaris* against Anthracnose disease caused by *C. lindemuthianum*	[57]
Ulvan	*U. fasciata*	Foliar spray	Stimulated resistance in *Arabidopsis* against *A. brassicicola* by increasing the activity of defense related antioxidant enzymes	[58]
Fucan	*Pelvetia canaliculata*	Leaf infiltration	Stimulated defense responses in tobacco against tobacco mosaic virus	[59]
Oligoulvans, oligoglucuronans	*U. lactuca*	Leaf infiltration	Reduced occurrence of wilt caused by *F. oxysporum* in tomato by inducing SA-dependent systemic acquired resistance	[60]
Glucuronan, oligoglucuronans	*U. lactuca*	In vitro assay	Reduced growth of *Penicillium expansum* and *B. cinerea* on apple fruit by modulating the generation of ROS and defense-related enzymes	[61]
oligo-sulphated-galactan	*Schyzimenia binderi*	Foliar spray	Enhanced activity of defense-related enzymes in tobacco against TMV infection	[18]
Eckol	*Ecklonia maxima*	Foliar spray	Increased aphid resistance in cabbage	[62]

^†^ The bioactive compounds used in these reports were purchased from commercial companies. The source of bioactive compounds was not mentioned.

**Table 2:** Roles of different extracts from various seaweeds in inducing disease resistance in different plants.

Extract	Source	Type of Extract	Mode of Application	Crop	Causal Organism	Disease	Function	References
Algamare^®^	*Ascophyllum nodosum*	Alkaline	Foliar spray	*Prunus salicina*	*Monilinia fructicola*	Brown rot	Reduced the incidence and severity of brown rot	[140]
Dalgin Active^®^	*A. nodosum*	Aqueous	Foliar spray	*Triticum aestivum, Triticum durum*	*Zymoseptoria tritici*	*Septoria tritici* blotch	Improved defense response by inducing expression of PR- proteins, antioxidant metabolism, and phenylpropanoid and octadecanoid pathways	[141]
Dalgin^®^	*A. nodosum*	Aqueous	Root drench	*Solanum lycopersicum*	*Phytophthora capsici*	Damping-off	Induced systemic defense response by eliciting the expression of defense-related genes or proteins	[142]
Marmarine^®^	*A. nodosum*	Alkaline	Foliar spray, root drench	*Cucumis sativus*	*Phytophthora melonis*	Damping-off	Control the progression of disease by inducing defense related enzymes	[139]
Maxicrop Original^®^	*A. nodosum*	Alkaline	In vivo assay	*Arabidopsis*	*Meloidogyne javanica*	Root-knot	Diminished population of females of *M. javanica* on treated plants	[136]
Maxicrop Triple^®^	*A. nodosum*	Alkaline	Foliar spray	*Fragaria × ananassa*	*Tetranychus urticae*	-	Control growth of the pest on treated plants	[143]
Stimplex^®^ (Acadian Seaplants)	*A. nodosum*	Alkaline	Foliar spray, root drench	*C. sativus*	*Alternaria cucumerinum, Didymella applanata, Fusarium oxysporum, Botrytis cinerea*	*Alternaria* blight, Gummy stem blight, *Fusarium* root and stem rot, *Botrytis* blight	Protect the plants by inducing the activity different-related enzymes and higher accumulation of secondary metabolites	[137]
Stimplex^®^	*A. nodosum*	Alkaline	Foliar spray	*S. lycopersicum, Capsicum annuum*	*Xanthomonas campestris* pv. *Vesicatoria, Alternaria solani*	bacterial spot, early blight	Reduced disease susceptibility by inducing the expression of defense responsive genes	[138]
Stella Maris^®^	*A. nodosum*	Alkaline	In vivo assay	*Arabidopsis thaliana*	*Pseudomonas syringae, P. aeruginosa, X. campestris*	-	Stimulated plant innate immunity by induction of stress-responsive genes.	[132]
Seasol^®^	*Durvillaea potatorum and A. nodosum*	Alkaline	Root drench	Broccoli	*Plasmodiophora brassicae*	Clubroot	Reduced the number of plasmodia formed in the root hairs	[144]
Seasol Commercial^®^	*D. potatorum, A. nodosum*	Alkaline	Root drench	*A. thaliana*	*Phytophthora cinnamomi*	-	Suppressed pathogen growth by the induction antioxidative defense pathways	[134]
*Ascophyllum nodosum* extract (Acadian Seaplants)	*A. nodosum*	Alkaline	Foliar Spray	Carrot	*Alternaria radicina* and *Botrytis cinerea*	Black rot, *Botrytis* blight	Confer immunity against pathogens by eliciting the expression of defense related genes or proteins	[133]
*A. nodosum* extract (Acadian Seaplants)	*A. nodosum*	Alkaline	Foliar spray, root drench	*S. lycopersicum*	*Alternaria solani, X. campestris pv vesicatoria*	*Alternaria* blight; Bacterial leaf spot	Protect plants by eliciting JA/ethylene dependent signalling pathways	[130]
*A. nodosum* extract (Acadian Seaplants)	*A. nodosum*	Alkaline and organic fractions	Root drench	*Arabidopsis*	*P. syringae*, *Sclerotinia sclerotiorum*	Bacterial speck, Stem rot	Controlled progression of diseases by in inducing the expression of JA-dependent signalling pathway	[145]
AMPEP (Acadian Seaplants)	*A. nodosum*	Alkaline	*-*	*K. alvarezii*	*Polysiphonia subtilissima*	Ice-ice, goose bumps	Controlled the epiphyte growth and showed reduce disease symptoms	[146,147]
AMPEP	*A. nodosum*	Alkaline	*-*	*K. alvarezii*	*Neosiphonia* sp.	Ice-ice	Improved the growth and reduce *Neosiphonia* infestation	[148]
AMPEP	*A. nodosum*	Alkaline	*-*	*K. alvarezii*	*Neosiphonia apiculata*	Ice-ice	Confer biotic stress tolerance against endophytes	[149]
*A. nodosum* extract	*A. nodosum*	Alkaline	Foliar spray	*Fragaria × ananassa*	*Podosphaera aphanis*	Powdery mildew	Reduced incidence and severity of powdery mildew by induction of defense related enzymes	[131]
Liquid Seaweed Extract (LSE)	*A. nodosum*	Alkaline	Foliar spray	*Triticum aestivum*	*Fusarium graminearum*	*Fusarium* head blight (FHB)	Increased resistance against FHB by inducing expression of defense responsive genes and enzymes	[150]
Kelpak^®^	*Ecklonia maxima*	Aqueous	Root drench	*C. annuum*	*Verticillium dahliae*	*Verticillium* Wilt	Reduced disease	[151]
Kelpak^®^-	*Ecklonia maxima*	Aqueous	Foliar spray, soil drench	*S. lycopersicum*	*Meloidogyne incognita*	-	Increased plant growth and lessened infestation	[152]
Kelpak^®^, OSMO^®^	*A. nodosum* and *Ecklonia maxima*	Aqueous, alkaline	Soil drench	*S. lycopersicum*	*Meloidogyne chitwoodi* and *Meloidogyne hapla*.	Root-knot	Reduced hatching, infectivity, and sensory perception of nematodes	[153]
K-sap	*Kappaphycus alvarezii*	Aqueous sap	Foliar spray	*S. lycopersicum*	*Macrophomina phaseolina*	charcoal rot	Reduced pathogen infestation by differentially regulating the expression of defense-related genes and phytohormone levels	[154]
*Ulva armoricana extract*	*U. armoricana*	Aqueous	Foliar spray	*P. vulgaris, Vitis Vinifera, Cucumis sativus*	*Erysiphe polygoni, E. necator, Sphareotheca fuliginea*	Powdery mildew	Protected plants against powdery mildew	[155]
Algal powder	*Gracilaria confervoides*	Dry powdered	Soil amendment	*C. sativus*	*Rhizoctonia solani, Fusarium solani, Macrophomina phaseolina*	-	Antifungal activity	[156]
Seaweed extracts	*Ulva lactuca, Caulerpa* *Sertularioides, Padina gymnospora, Sargassum liebmannii*	Aqueous	Soil drench, foliar spray	*S. lycopersicum*	*Alternaria solani*	Early blight	Reduced necrotic lesion	[157]
Seaweed extracts	*Ulva lactuca, Sargassum filipendula* and *Gelidium serrulatum*	Alkaline	Foliar spray	*S. lycopersicum*	*Alternaria solani and Xanthomonas campestris pv vesicatoria*	Early blight, bacterial spot	Reduced disease severity by inducing the activities of defense enzymes and expression of genes involved defense signalling pathways	[158]
*U. lactuca* extract	*U. lactuca*	Aqueous	In vitro assay	*Malus domestica*	*Penicillium expansum* and *Botrytis cinerea*	Blue and gray mould	Reduced the lesion by activating antioxidant-related enzyme and phenylpropanoid metabolism	[105]
*U. lactuca* extract	*U. lactuca*	Aqueous, organic fractions	In vitro assay	*Citrus sinesis*	*Penicillium digitatum*	Citrus green mold	Reduced spore germination	[159]
*Ulva* extract	*Ulva* spp.	Aqueous	Foliar spray	*Medicago truncatula*	*Colletotrichum trifolii*	-	Elicited the defense response by inducing the expression of defense-related gene	[160]
Seaweed extracts	*Cystoseira myriophylloides, Laminaria digitata and* *Fucus spiralis*	Aqueous	Foliar spray	*Nicotiana benthamiana*	*Pseudomonas syringae* pv. *tabaci*	wildfire	Controlled the progression of wildfire disease by inducing antioxidant defenses	[161]
-	*S. tenerrimum, S. wightii, S. swartzii*	Dry powder	Soil amendment	*Helianthus annuus*	*Macrophomina phaseolina, F. solani,*	Root rot disease	Controlled the progression of disease in plants	[162]
S-extract	*S. tenerrimum*	Aqueous	Foliar spray	*S. lycopersicum*	*Macrophomina phaseolina*	Charcoal rot	Stimulated plant defenses by regulating antioxidative and phytohormone metabolism	[163]
*S. vulgare* extracts	*S. vulgare*	Aqueous, organic fractions	In vitro assay	*Solanum tuberosum*	*Pythium aphanidermatum*	*Pythium* leak	Antifungal activity against pathogen	[164]
*S. vulgare* extracts	*S. vulgare*	Aqueous, organic fractions	In vitro assay	*Solanum tuberosum*	*Fusarium oxysporum f. sp. tuberosi*	*Fusarium* Dry Rot	Controlled the progression of disease in tubers	[165]
Seaweed extract	*Sargassum polycystum*	Aqueous	Foliar spray	*Hevea brasiliensis*	*Phytophthora palmivora*	Leaf fall	Foliar spray confers resistance by inducing systemic acquired resistance triggered enzymes and anti-oxidative defense enzymes	[166]
Sea algal product	*Sargassum fusiforme*	Aqueous	Foliar spray	*S. lycopersicum*	*P. infestans; B. cinerea; Odium sps.*	Late blight, grey mold, powdery mildew	Foliar spray controlled the progression of late blight, grey mold and powdery mildew	[167]
-	*S. vulgare, Acanthophora spicifera*	Alkaline	Foliar spray	*S. lycopersicum, Capsicum annum*	*A. solani and X. campestris*	Early blight, bacterial spot	Induced defense by regulation of expression of genes involved in defense-response and phytohormone biosynthesis	[168]
-	*Turbinaria conoides*	Aqueous, alcoholic	In vivo assay	*-*	*Fusarium oxysporum*	Root rot	Possess antifungal activity	[169]
Seaweed extracts	*Cystoseira myriophylloides*, *Laminaria digitata*, and *Fucus spiralis*	Aqueous	Foliar spray	*S. lycopersicum*	*Verticillium dahliae, Agrobacterium tumefaciens*	*Verticillium* wilt of tomato, Crown gall	Induced plant defense by increased activity of defense-related enzymes	[170]

“-” This represents that the particular literature cited is devoid of the information mentioned in the column.
